# Supplementary material for: Vortex entropy and superconducting fluctuations in ultrathin underdoped Bi2Sr2CaCu2O8+x superconductor
Source: Nat Commun. 2024 Jun 6;15:4818. doi: 10.1038/s41467-024-48899-6 (PMC11156657; doi:10.1038/s41467-024-48899-6)
Supplement: Supplementary file 1 — Supplementary information [file 41467_2024_48899_MOESM1_ESM.pdf]

Supplementary information to:

Vortex entropy and superconducting fluctuations in ultrathin underdoped $Bi_2Sr_2CaCu_2O_{8+x}$ superconductor

Shuxu Hu[1], Jiabin Qiao[1,2,3*], Genda Gu[4], Qi-Kun Xue[1,3,5,6*], and Ding Zhang[1,3,6,7*]

[1]State Key Laboratory of Low Dimensional Quantum Physics and Department of Physics, Tsinghua University, Beijing 100084, China

[2]Centre for Quantum Physics, Key Laboratory of Advanced Optoelectronic Quantum Architecture and Measurement, School of Physics, Beijing Institute of Technology, Beijing 100081, China

[3]Beijing Academy of Quantum Information Sciences, Beijing 100193, China

[4]Condensed Matter Physics and Materials Science Department, Brookhaven National Laboratory, Upton, NY 11973, USA

[5]Southern University of Science and Technology, Shenzhen 518055, China

[6]Frontier Science Center for Quantum Information, Beijing 100084, China

[7]RIKEN Center for Emergent Matter Science (CEMS), Wako, Saitama 351-0198, Japan

*Email: jiabinqiao@bit.edu.cn, qkxue@mail.tsinghua.edu.cn, dingzhang@mail.tsinghua.edu.cn

Contents

Supplementary Note 1. Thermal activation of bulk-like BSCCO

Supplementary Note 2. Nernst signal of thick BSCCO flakes

Supplementary Note 3. Seebeck effect in ultrathin BSCCO

Supplementary Note 4. Linear response in the transport measurement

Supplementary Note 5. Criteria for defining T_c

Supplementary Note 6. Onset temperature for superconducting fluctuations

Supplementary Note 7. Gaussian superconducting fluctuations

Supplementary Figures 1-15

Tables S1-S3

Supplementary Note 1. Thermal activation of bulk-like BSCCO

Vortices in a multilayer system tend to align along the c-axis and form flux lines. The thermal activation mainly deforms these flux lines [1]. For the ultrathin or monolayer superconductors, however, the thermal activation is governed by the collective creeping of vortices in 2D. This latter behavior was observed previously in $YBa_2Cu_3O_y/PrBa_2Cu_3O_y$ (YBCO/PBCO) superlattices [2,3]. However, the presence of dimensionality crossover remained an open question.

In thick flakes of BSCCO, we observe a linear trend in the double logarithmic plot (Supplementary Fig. 4b), indicating a power law dependence: $U \propto B^{-\alpha}$, where α is a fitting parameter. This power law behavior is consistent with previous studies on bulk BSCCO [4,5]. It is qualitatively different from that observed in ultrathin samples (See Fig. 2h and Supplementary Fig. 4d). This sharp contrast reflects a drastic change in the vortex dynamics.

Supplementary Note 2. Nernst signal of thick BSCCO flakes

Samples S6 to S9 are relatively thick BSCCO flakes. S6 to S7 were exfoliated from OP bulk crystal and S8-S9 were from an underdoped bulk crystal. The Nernst trace $N(B)$ of each sample rises with increasing temperatures at first due to vortex melting and gets attenuated at higher temperatures as the superconductivity disappears. Such a temperature dependence of $N(B)$ basically resembles that of ultrathin BSCCO and yields a peak profile at a fixed B (Supplementary Fig. 5b1-b4). As shown in Supplementary Fig. 5b1, the Nernst peak N_p of S6 is quantitatively consistent with that of ultrathin sample. The data are also in agreement with previous measurements on bulk crystals [6]. The peak position T_{Np} shares the same doping evolution as ultrathin BSCCO. In nearly OP samples of S6 and S7, T_{Np} is slightly lower than T_c by 5-10 K. By contrast, T_{Np} becomes higher than T_c in underdoped samples of S8 and S9.

Supplementary Note 3. Seebeck effect in ultrathin BSCCO

Supplementary Figure 6a displays the thermopower (Seebeck coefficient) $S(T)$ from samples S1 to S5. The general temperature dependence—a broad peak at around 100 K to 150 K with a linear decreasing trend at higher temperatures—is consistent with previous studies [7,8]. In Supplementary Fig. 6b, we summarize the evaluated S at 250 K for all the samples. They fall on an exponential decay with p (solid straight line), consistent with previous reports [7].

Supplementary Note 4. Linear response in the transport measurement

In Supplementary Fig. 7a, we investigate an underdoped sample (S9) in the thermally activated flux flow regime. We observe that the longitudinal voltage V_{xx} is linear with the input ac current I down to 0.1 uA (See Supplementary Fig. 7a). We show in Supplementary Fig. 7b that the transverse voltage δV_{xy} induced by the vortex Nernst effect is also linear with the temperature difference δT_{xx} in the operating range. These results confirm that our electrical and thermoelectric measurements are carried out in a linear regime.

Supplementary Note 5. Criteria for defining T_c

In the main text, we define T_c as the temperature where the resistivity drops to 1% of normal state resistivity at zero magnetic field. This is close to the zero-resistance temperature as employed previously for analyzing the superconducting fluctuations. For defining T_c from resistivity data of a superconductor, the criterion of using 50% or 90% ρ_n is also widely employed. We show in Supplementary Fig. 9 these different definitions.

We now compare the different criteria with the Nernst signals. Supplementary Figure 10 provides a 2D colored plot of N as a function of B and T. The temperature points where $\rho_n(B)$ of the same sample drops to 1%, 50%, and 90% of $\rho_n(B=0)$ are overlaid on the colored plot. For S1 to S4 as well as the thick sample S7, the trend of $T_c^{1\%}(B)$ matches well with the contour of N at the low temperature side. It suggests that at $T < T_c^{1\%}(B=0)$, the resistivity starts to rise as a function of magnetic field mainly due to the flux flow or melting of vortex lattice. The region below the $T_c^{1\%}(B)$ curve at lower temperature and magnetic fields corresponds to the vortex lattice regime. The curve of $T_c^{50\%}(B)$ almost overlaps with the large Nernst signal (dark red). It indicates that flux flow dominates at this temperature [below $T_c^{1\%}(B=0)$] and magnetic field. However, we do not see direct correspondence between the $T_c^{50\%}(B)$ or $T_c^{90\%}(B)$ curve with the contour of N. This is possibly due to the mixed contribution to resistivity from both vortex motion and quasi-particles, whereas the vortex motion dominates the contribution to the Nernst signal at $T < T_c^{1\%}(B=0)$.

Supplementary Note 6. Onset temperature for superconducting fluctuations

The contribution of quasi-particles to the Nernst effect usually has a linear dependence on the magnetic field: $N \propto B$. As the temperature decreases and approaches T_c, the Nernst signal can behave non-linearly with B due to the emergence of superconducting fluctuations. Cyr-Choinière *et al.* [9] analyzed the Nernst coefficient $\nu = N/B$ and defined the onset for superconducting fluctuations—T_b—as the temperature where the values of ν at different B no longer overlap, i.e., a nonlinear behavior of N as a function of B. To quantify this analysis, here we use the following protocol. First, we evaluate the sampling standard deviation σ_N of the $N(B)$ trace at T_{cr}, where dN/dB is nearly zero. Since the Nernst signal itself should also be close to zero at this temperature, σ_N mainly reflects the noise level of our measurement. Secondly, we subtract the $N(B)$ trace by a straight line that connects zero point and $N(B = B_{max})$ (dashed lines in Supplementary Fig. 11a1-a5). We again evaluate the sampling standard deviation of the subtracted curve: σ_N^{sub}. These values are plotted in Supplementary Fig. 11b1-b5. We define T_b as the temperature point where σ_N^{sub} exceeds the experimental noise level of σ_N. It indicates that the extent of nonlinearity in the Nernst trace at $T < T_b$ becomes clearly noticeable.

Supplementary Note 7. Comparison with Gaussian superconducting fluctuations

Here we analyze the data of S3 to S5 above T_c (The dataset of S1 and S2 is too limited in the regime of superconducting fluctuations) by using the theory of Gaussian superconducting fluctuations (GSF) [10,11]. In a layered superconductor, the theoretically derived off-diagonal Peltier coefficient in a small magnetic field is [10]:

$$\alpha_{xy}^{GSF} = \frac{k_B e^2 B \xi_{ab}^2}{6\pi\hbar^2 s}\frac{1}{\sqrt{1+(2\xi_c/s)^2}}, \quad \text{(S1)}$$

where s is the interlayer spacing (1.5 nm for BSCCO), ξ_{ab} (ξ_c) is the in-plane (c-axis) coherence length. The temperature dependence of $\xi_{ab,c}$ is described by $\xi_{ab,c}(T) = \xi_{ab}^{(0)}/\sqrt{t}$ and $t = (T - T_c)/T_c$. Since $\xi_c \ll s$ in BSCCO, we can neglect the term related to ξ_c and obtain:

$$\alpha_{xy}^{GSF}/B = \frac{k_B e^2 (\xi_{ab}^0)^2}{6\pi\hbar^2 s} t^{-1}. \quad \text{(S2)}$$

By using the definition $\alpha_{xy}/B = N/(\rho B) = \nu/\rho$, Eq. (S2) indicates that the Nernst coefficient ν at a fixed t is a constant in the zero-field limit.

Supplementary Fig. 12a1-a3 show the experimentally measured ν of S3-S5 in a log-log plot. At small magnetic fields, ν shows a plateau-like behavior that seems consistent with that predicted by GSF. We note that similar plateau behaviors were reported in the previous studies on superconducting fluctuations in Nb_xSi_{1-x} [12], Eu-LSCO [13] and PCCO [14]. By using the data above the noise level σ_N/B (σ_N is defined in the previous note), we evaluate ν in the zero-field limit (ν_0) by averaging over the plateau region (horizontal dashed lines in Supplementary Fig. 12a1-a3). The obtained values further divided by the zero-field resistivity are plotted as a function of t in Supplementary Fig. 12b1-b3. At higher temperature points, the experimental trend is much faster than $1/t$, indicating that the contribution from quasi-particles starts to take over. We also note that at T too close to T_c there exists no plateau behavior in the logarithmic plot (for example, $T = 10$ K for S5), suggesting that GSF is not applicable here.

In an intermediate temperature range, the extracted ν_0/ρ_0 shows a $1/t$ dependence. Apart from the slope, Eq. (S2) suggests a single fitting parameter—ξ_{ab}^0

to describe ν_0/ρ_0 as a function of t. From magneto-transport, we estimate the superconducting coherence length as 1.6, 3.3, and 6 nm for S3 to S5 (We take the intercepts in the magnetic field axis in the $U(B)$ plot of Fig. 2h as the upper critical field B_{c2}^0 and then obtain the coherence length by using $\xi_{ab}^0 = \sqrt{\Phi_0/(2\pi B_{c2}^0)}$). In Supplementary Fig. 12b1-b3, the dashed lines are obtained by inserting these values into Eq. (2). For sample S5, the dashed line nicely captures the experimental trend. For the other two samples, the disagreement can be mitigated by adjusting the coherence length values (5.5 nm for S3 and 2 nm for S4).

As a final remark, we also point out that the quasi-particle contribution to the Nernst signal in the regime just above T_c should be negligible. First of all, the quasi-particle contribution is controlled by the mean free path [15, 16]. The mean free paths in our ultrathin samples are in general shorter than those of bulk samples. To illustrate this point, we estimate the mean free path l by using [17]:

$$l = \frac{\sigma_0/\frac{e^2}{h}}{\sqrt{2\pi n_{2D}}},$$

where σ_0 is the normal state conductivity of each BSCCO monolayer (half a unit cell), n_{2D} is the two-dimensional carrier density. We estimate n_{2D} via $n_{2D} = 2p/a^2$, where a is the in-plane lattice constant. Supplementary Figure 13 shows the estimated mean free paths of our samples. They show values that are much smaller than those in $Pr_{2-x}Ce_xCuO_4$ (PCCO) [14]. We also estimate the quasi-particle contribution by using $S \cdot tan\,\theta\,/B$ and compare it with the Nernst coefficient ν_0 in the regime of superconducting fluctuations. Table S3 shows that $S \cdot tan\,\theta\,/B$ is two orders of magnitude smaller than ν_0.

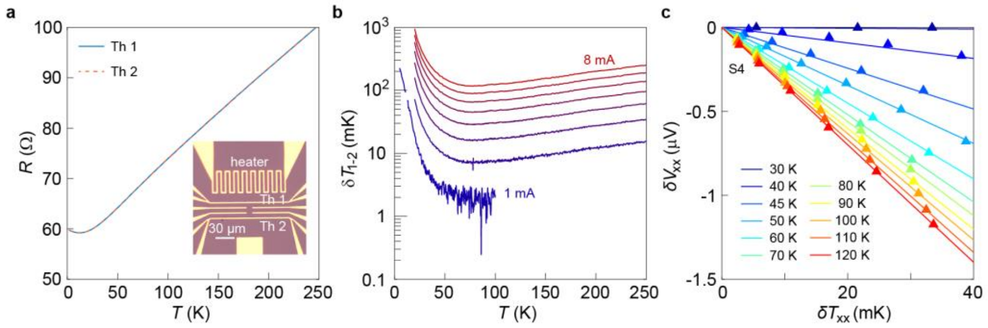

Supplementary Figure 1. Calibration of the on-chip thermometry. **a**, Resistance of the two thermometers (Th1, Th2 in the inset) as a function of bath temperature T. The inset shows an optical image of the electrodes. **b**, Temperature difference between the thermometers δT_{1-2} under different heating power ($I_{ac} = 1, 2, \cdots 8\ \mathrm{mA}$) as a function of T. **c**, Thermopower voltage δV_{xx} of sample S4 as a function of δT_{xx} at different T.

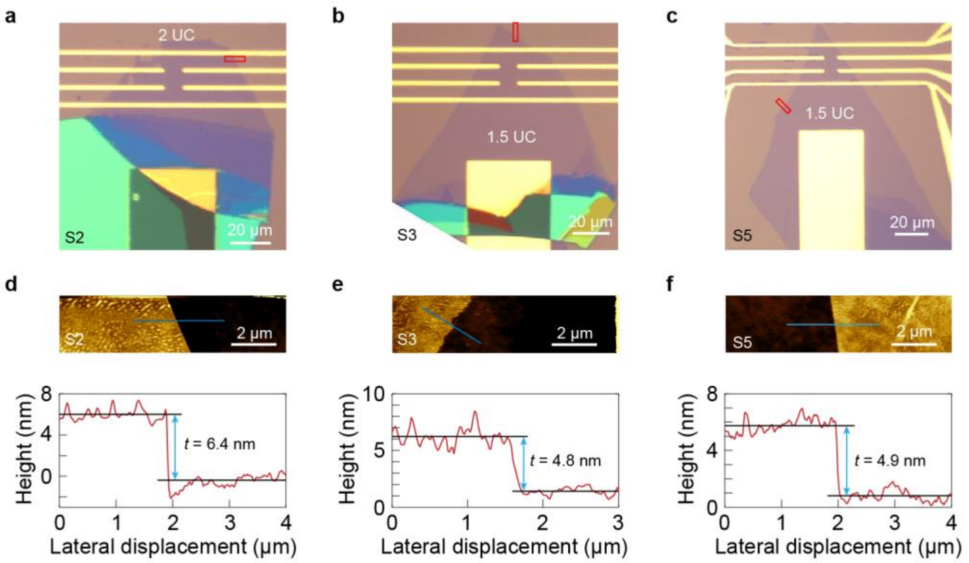

Supplementary Figure 2. Thickness characterization of ultrathin BSCCO. **a-c**, Optical images of samples S2, S3, and S5 before capping h-BN. The red rectangles indicate the scanning regions for atomic force microscopy (AFM) after the transport measurements. **d-f**, AFM images (top) and line profiles (bottom) of samples S2, S3, and S5. The apparent step height is indicated by the double arrow.

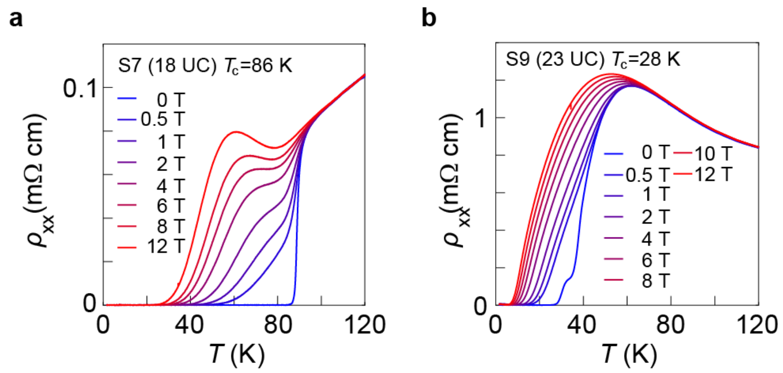

Supplementary Figure 3. Resistivity in bulk-like BSCCO samples. **a, b**, Temperature dependent resistivity of S7 and S9 at different perpendicular magnetic fields.

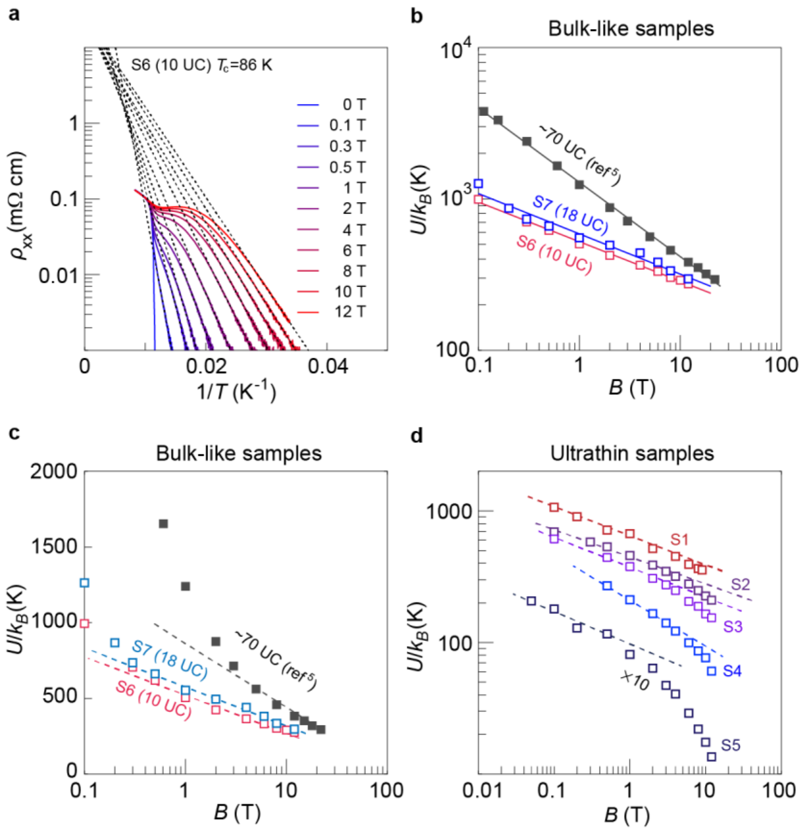

Supplementary Figure 4. Activation energy in bulk-like BSCCO. **a**, Arrhenius plot of the temperature dependent resistivity of S6 at different magnetic fields. Dashed lines are linear fits. **b**, Activation energy U plotted as U/k_B as a function of B for relatively thick BSCCO in a log-log plot. Filled data points are digitized from ref [5]. Solid lines are linear fits. **c**, U/k_B as a function of B for bulk-like samples in a semi-log plot. **d,** U/k_B as a function of B for ultrathin samples in a log-log plot. The dashed lines in panel **c** and **d** are guides to the eye.

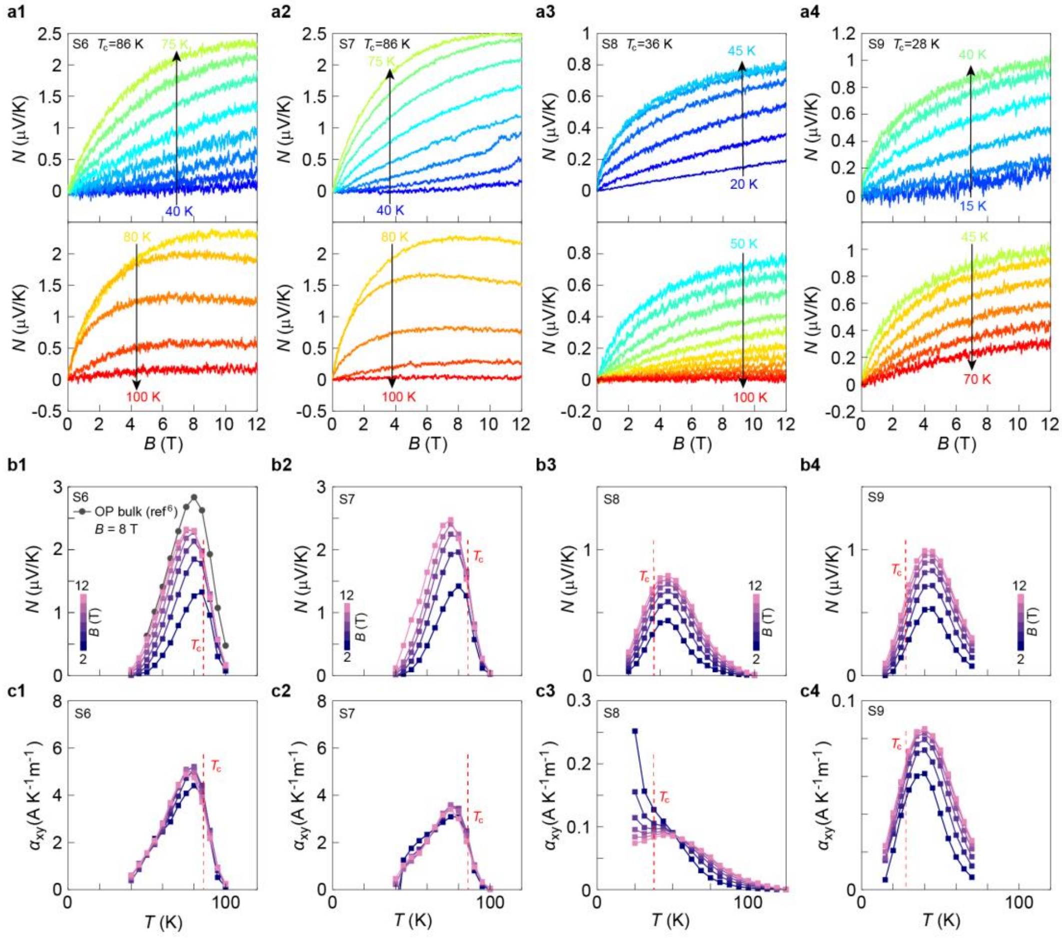

Supplementary Figure 5. Nernst signals and off-diagonal Peltier coefficients in bulk-like BSCCO samples. **a1-a4**, Nernst signals N of S6-S9 as a function of B measured at different temperatures. The temperature step between neighbor curves is 5 K. The direction of the black arrow indicates the evolution of the Nernst traces as temperatures increase. **b1-b4**, Nernst signals of S6-S9 as a function of temperatures at fixed B (For S6, S8, S9, $B = 2, 4, 6, 8, 10, 12$ T; For S7, $B = 2, 4, 6, 8, 12$ T). The red dashed lines mark the position of T_c. We also show data of the bulk BSCCO in panel **b1** for comparison. **c1-c5**, Off-diagonal Peltier coefficients $\alpha_{xy} = N/\rho_{xx}$ as a function of temperature at fixed B (Same color coding as panel **b1**-**b4**).

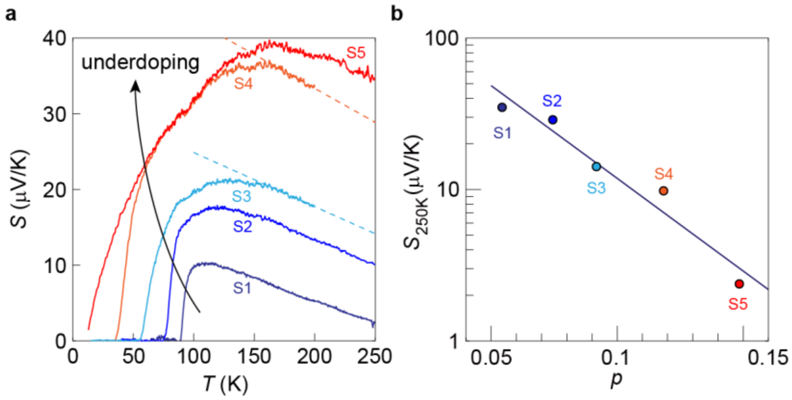

Supplementary Figure 6. Thermopower of ultrathin BSCCO. a, Seebeck coefficient S as a function of T in ultrathin BSCCO samples (S1-S5). The dashed lines are linear extrapolations. **b**, Seebeck coefficients at 250 K as a function of the doping level p. The solid line is a linear fit. For samples S3 and S4, we linearly extrapolate S to 250 K (dashed lines in panel **a**).

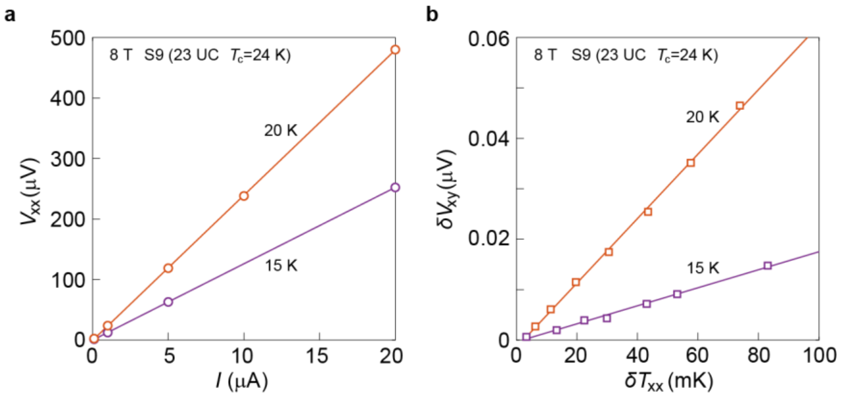

Supplementary Figure 7. Examination of the linear response in resistivity and Nernst signal measurements. **a**, Longitudinal voltage V_{xx} as a function of the input current I in the resistivity measurement. **b**, Transverse voltage δV_{xy} as a function of the temperature difference δT_{xx} in the Nernst signal measurement. Straight lines are linear fits.

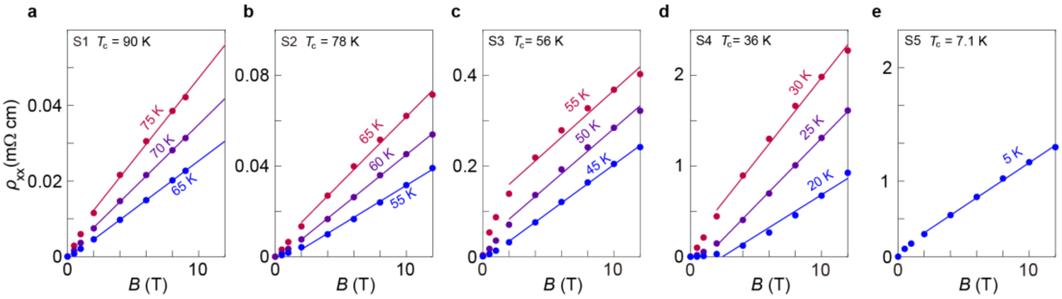

Supplementary Figure 8. Magneto-resistivity at selected temperatures around the corresponding Nernst peak. **a-e**, Resistivity of S1-S5 as a function of B below T_c. Solid lines are linear fit for resistivity data above 2 T. Linear behavior suggests that the transport is in the flux flow regime.

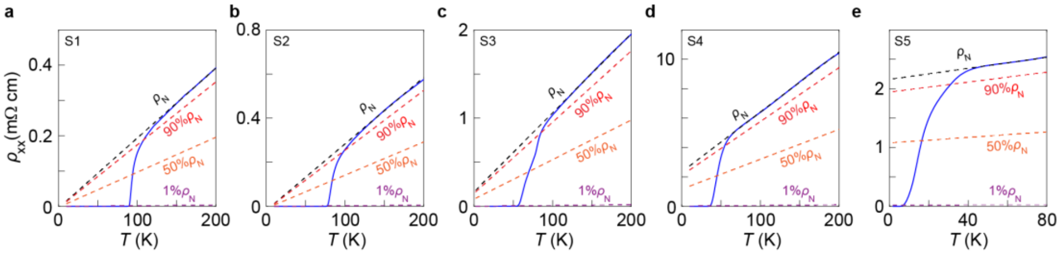

Supplementary Figure 9. Different criteria for defining T_c. a-e, resistivity data of samples S1 to S5. Black dashed lines are linear extrapolations showing the normal state resistivity ρ_N. Purple/orange/red dashed lines represent the lines for 1%, 50% and 90% of ρ_N, respectively.

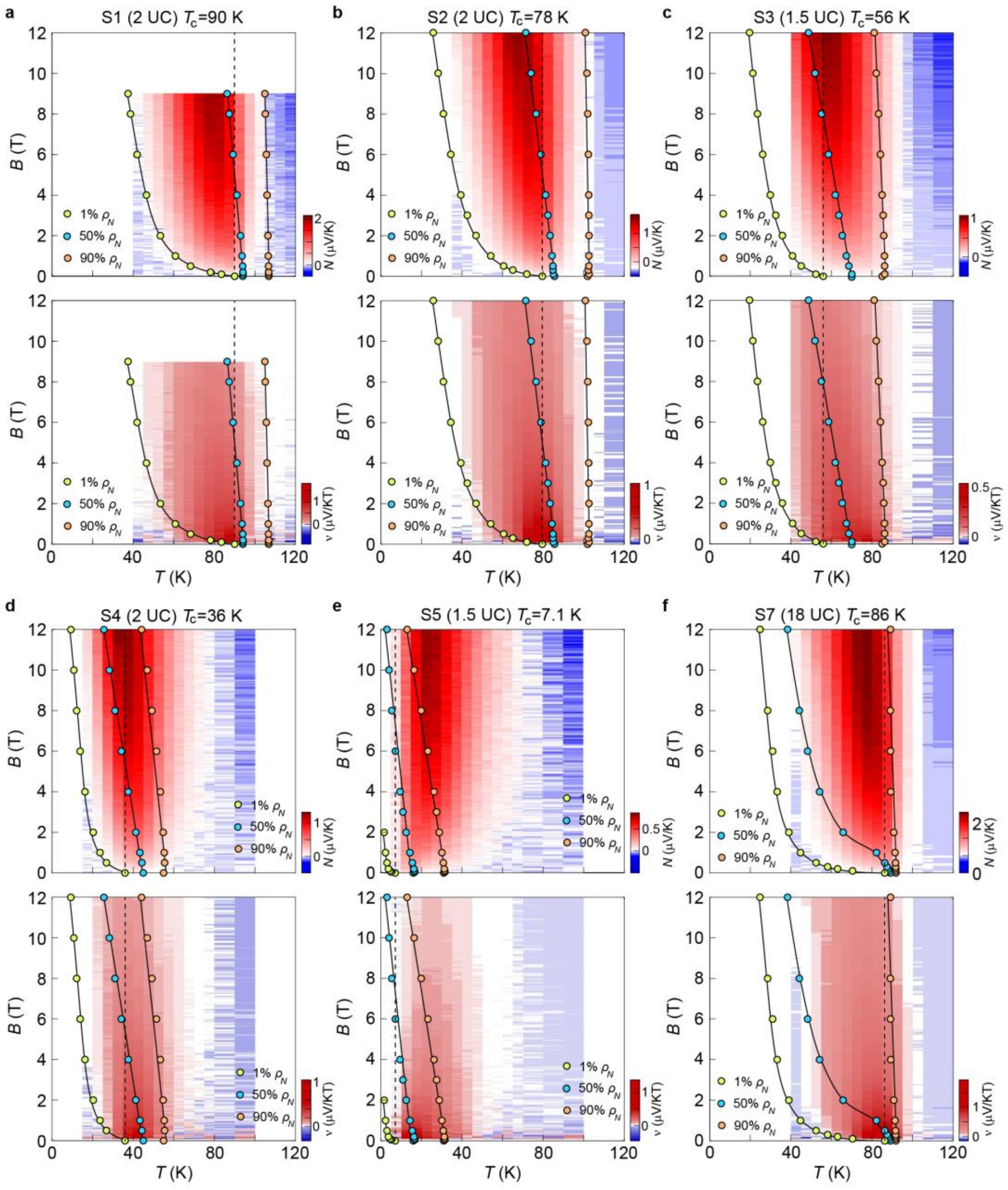

Supplementary Figure 10. Color rendition of Nernst signals. **a**-**e**, N (upper panels) and $\nu = N/B$ (lower panels) as a function of T and B in ultrathin samples. **f**, N (upper panel) and ν (lower panel) as a function of T and B in thick sample. Circles mark the temperature where the resistivity at certain B drops to 1%, 50% and 90% of normal state resistivity ρ_N. The dashed line marks the position of $T_c{}^{1\%}$ at the zero field. The solid lines are guides to eyes.

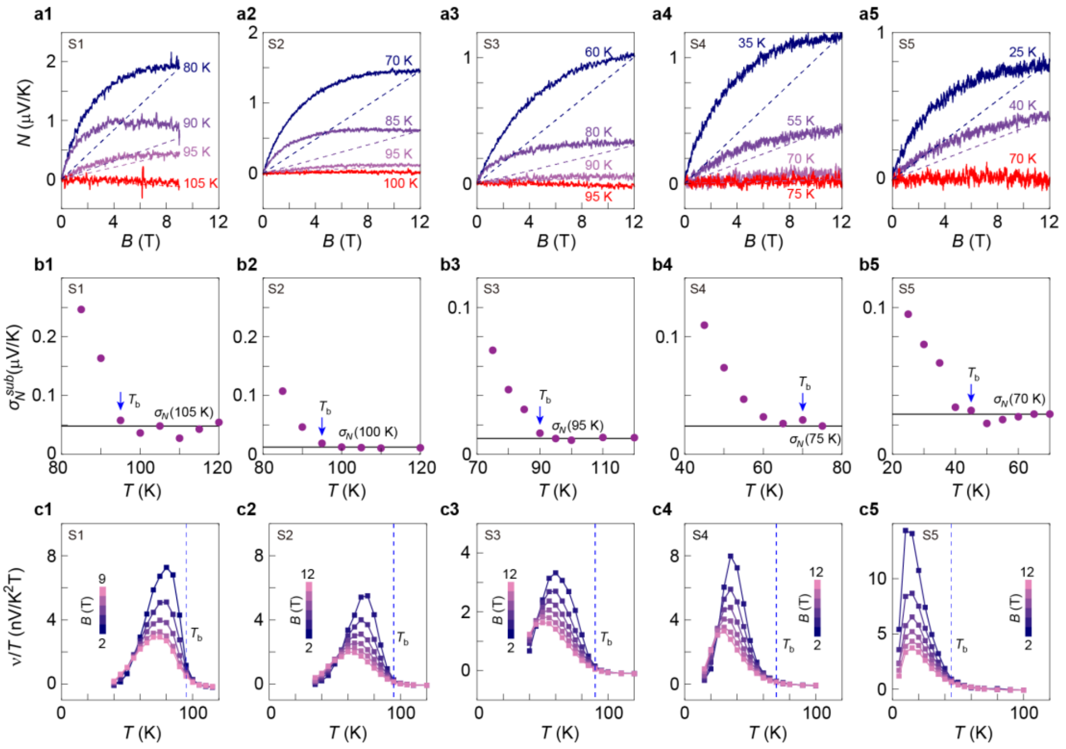

Supplementary Figure 11. Determination for the onset temperature of superconducting fluctuations. **a1-a5**, Nernst signals of S1-S5 as a function of B above T_{cr}. The dashed line in each panel connects the zero point and $N(B = B_{max})$. **b1-b5**, Sampling standard deviation σ_N^{sub} of the subtracted Nernst signals as a function of T for S1-S5. Black line in each panel marks the level of sampling standard deviation σ_N of the $N(B)$ trace at T_{cr}. Blue arrows mark the positions of T_b. **c1-c5**, Nernst coefficient $\nu = N/B$ plotted as ν/T versus T at selected B (For S1, $B = 2, 4, 6, 8, 9$ T; For S2-S5, $B = 2, 4, 6, 8, 10, 12$ T). The dashed lines mark the positions of T_b determined in panel **b1-b5**.

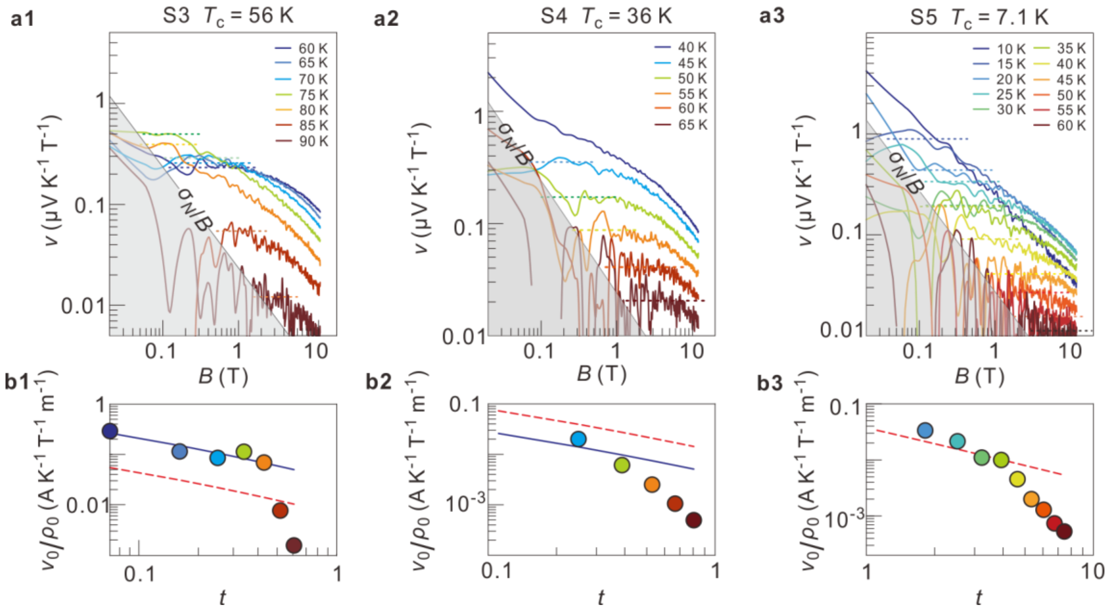

Supplementary Figure 12. Comparison with Gaussian superconducting fluctuations. **a1-a3**, Nernst coefficient ν as a function of magnetic field B at temperature points above T_c in ultrathin samples S3-S5, respectively. The dashed lines mark the plateau regions where ν keeps a constant. The gray solid lines denote the sampling standard deviation of Nernst coefficient (σ_N/B). The shaded gray triangles reflect that the data in such regions are in the noise level of our measurements. **b1-b3**, Nernst coefficient in the zero-field limit, ν_0, divided by the zero-field resistivity ρ_0 in **a1-a3** as a function of the reduced temperature $t(= T/T_c - 1)$, respectively. The dashed lines are obtained by inserting the extracted coherence length ξ_{ab}^0 (1.6, 3.3, and 6 nm for S3, S4 and S5) from magneto-transport into Eq. (2). The solid lines in **b1** and **b2** are obtained by adjusting the coherence length values in S3 (5.5 nm) and S4 (2 nm), respectively.

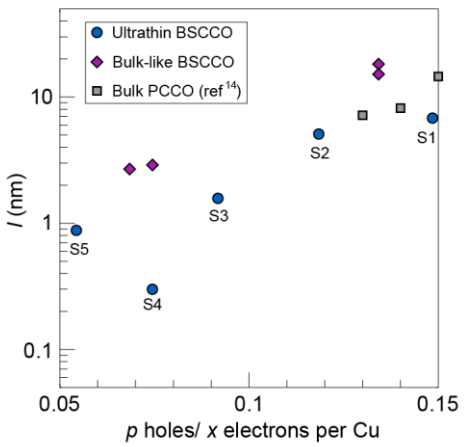

Supplementary Figure 13. Mean free path in BSCCO samples. Circles, diamonds represent the mean free paths for ultrathin and bulk-like BSCCO respectively. For comparison, the mean free path of $Pr_{2-x}Ce_xCuO_4$ (PCCO) from ref[14] is included.

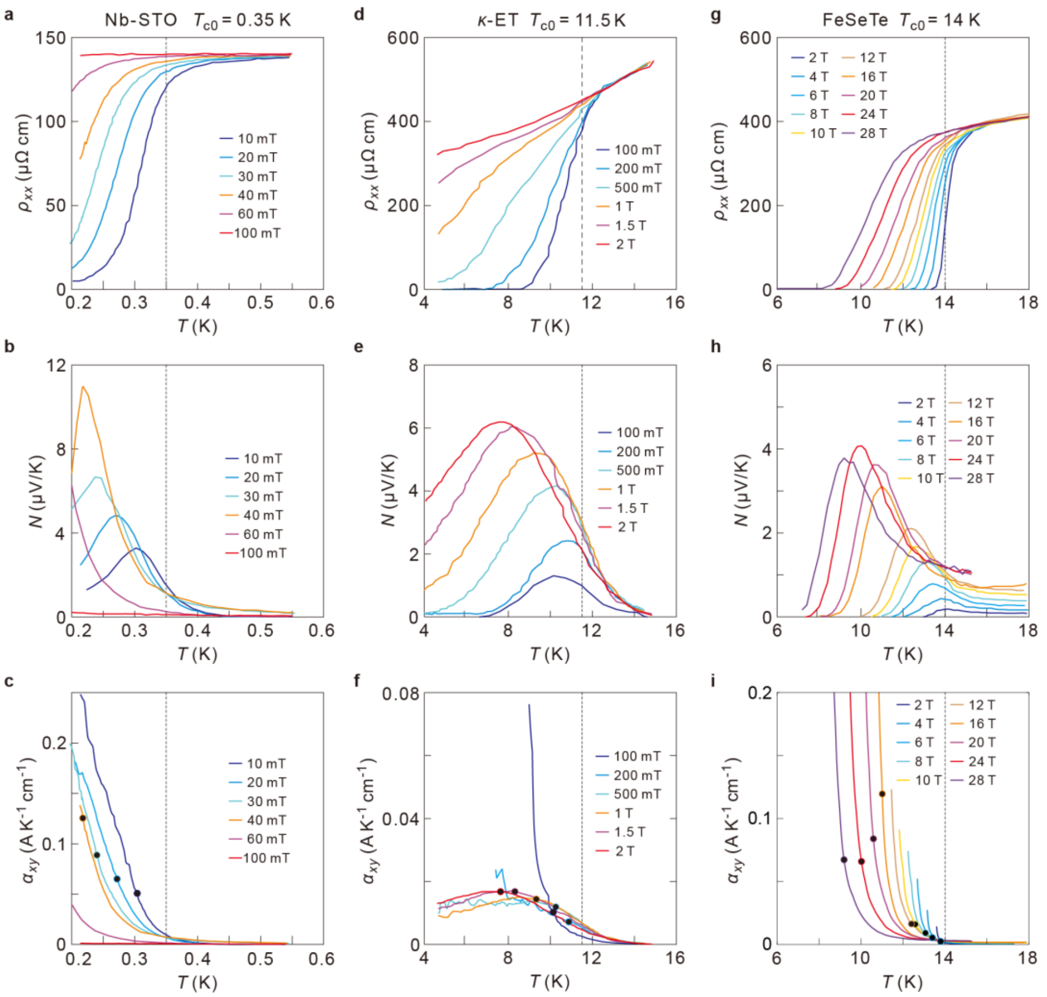

Supplementary Figure 14. a-i, Temperature dependence of resistivity ρ_{xx} (**a, d, f**), Nernst signal N (**b, e, h**) and off-diagonal Peltier coefficient α_{xy} (**c, f, i**) in STO, κ-ET and FeSeTe, respectively. Insets in panels **a**, **b** and **c** show ρ_{xx}, N and α_{xy} in STO as a function of magnetic field B at different temperatures. Dotted lines mark T_c. Black dots mark temperature points of Nernst peaks at selected magnetic fields.

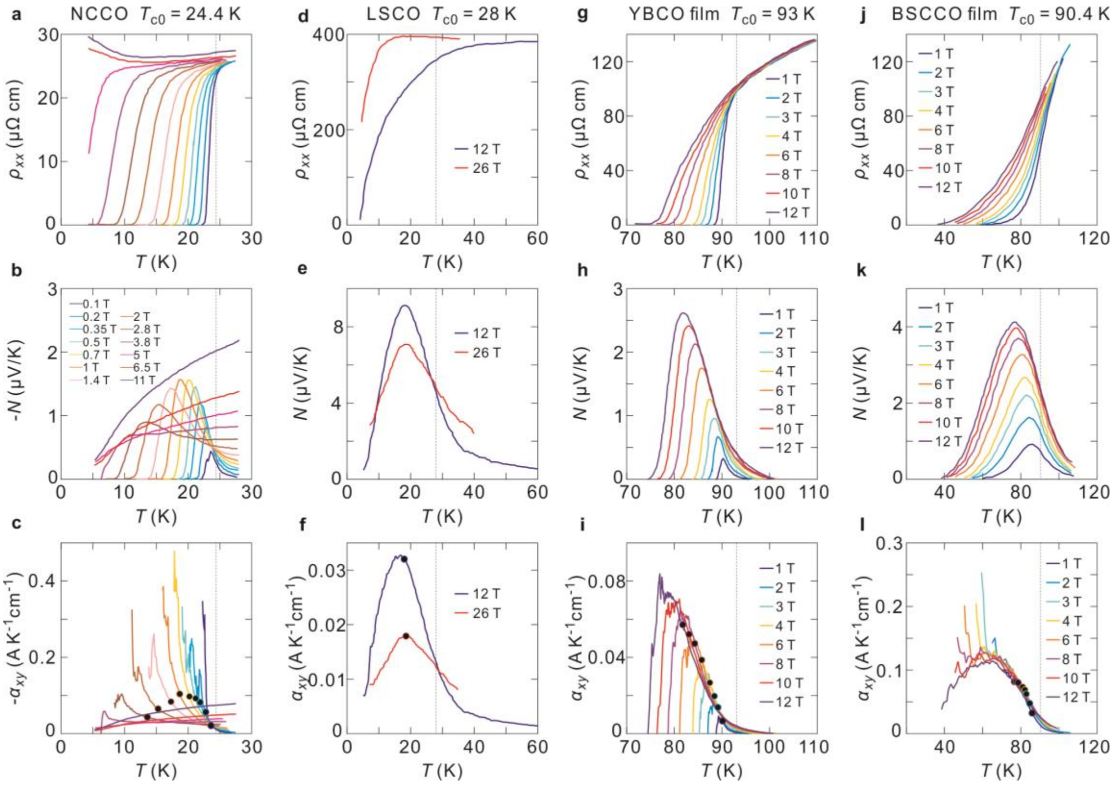

Supplementary Figure 15. a-l, Temperature dependence of resistivity ρ_{xx}, Nernst signal N and off-diagonal Peltier coefficient α_{xy} in NCCO, LSCO, epitaxially grown films of YBCO and BSCCO. Dotted lines mark T_c. Black dots mark temperature points of Nernst peaks at selected magnetic fields.

Table S1 London penetration depth in BSCCO at (nearly) optimal doping level. The corresponding plot is shown in Fig. 2j (See the main text).

λ (λ_{ab}) (nm)	T_c (K)	Technique	Reference
260	91	Microwave surface impedance	Ref. [18]
270	--	Lower critical field H_{c1} measurements	Ref. [19]
210	85	Reversible magnetization	Ref. [20]
180	84	µSR	Ref. [21]
125	90	Thermally activated transport measurements	Our work

Table S2 Parameters used in the calculation of vortex entropy in different superconductors. The corresponding plot of vortex entropy is shown in Fig.4 (See the main text).

Materials	T_c (K)	T^{peak} (K)	B^{peak} (T)	N^{peak} (μV/K)	ρ^{peak} ($\mu\Omega$ cm)	c (nm)	S_d^{sheet} (10^{-23}J/K)	Comments
STO	0.35	0.21	0.04	11	100	0.39	0.89	Ref. [22] Supplementary Fig. 14(a-c)
$Nb_{80}Mo_{20}$	4.15	3.5	0.01	--	--	0.3	300	Ref. [23]
$NbSe_2$	4.18	3	0.5	5	55	0.6	2.82	Ref. [24]
κ-ET	11.5	7.61	2	6.1	380	2.9	0.96	Ref. [25] Supplementary Fig. 14(d-f)
FeSeTe	14	10	24	4	48	0.58	0.96	Ref. [26] Supplementary Fig. 14(g-i)
NCCO	24.4	18.7	1	1.6	20	1.2	1.9	Ref. [27] Supplementary Fig. 15(a-c)
LSCO	29	18.5	12	9.1	260	1.2	0.88	Ref. [28] Supplementary Fig. 15(d-f)
YBCO film	93	81.8	12	2.6	50	1.19	1.3	Ref. [29] Supplementary Fig. 15(g-i)
BSCCO film	90.4	76.8	12	4.1	50	3.1	5.1	Ref. [29] Supplementary Fig. 15(j-l)
YBCO crystal	90	77	10	--	--	1.19	5.47	Ref. [30]

Table S3 Magnitude of $\boldsymbol{S}\tan\boldsymbol{\theta}/\boldsymbol{B}$ and the Nernst coefficient $\boldsymbol{\nu_0}$ in the zero-field limit for sample S3-S5. $S\tan\theta/B$ is evaluated at 100 K and 1 T. The values of ν_0 are taken in the temperature window where ν_0/ρ_0 shows a $1/t$ dependence as shown in Supplementary Fig. 12.

	$\frac{S\tan\theta}{B}$ (nV/K T)	ν_0 (nV/K T)
S3	3.04	200-500
S4	5.87	150-400
S5	6.47	200-900

References

[1] Clem, J. R. Two-dimensional vortices in a stack of thin superconducting films: A model for high-temperature superconducting multilayers. *Phys. Rev. B* **43**, 7837 (1991).

[2] Brunner, O., Antognazza, L., Triscone, J.-M., Miéville, L. & Fischer, Ø. Thermally activated flux motion in artificially grown $YBa_2Cu_3O_7/PrBa_2Cu_3O_7$ superlattices. *Phys. Rev. Lett*. **67**, 1354 (1991).

[3] Yang, H. C., Wang, L.M. & Horng, H.E., Characteristics of flux pinning in $YBa_2Cu_3O_y/PrBa_2Cu_3O_y$ superlattices. *Phys. Rev. B* **59**, 8956 (1999).

[4] Palstra, T. T. M., Batlogg, B., Schneemeyer, L. F. & Waszczak, J. V. Thermally activated dissipation in $Bi_{2.2}Sr_2Ca_{0.8}Cu_2O_{8+\delta}$. *Phys. Rev. Lett*. **61**, 1662 (1988).

[5] Zhang, Y. Z., Wang, Z., Lu, X. F., Wen, H. H., de Marneffe, J. F., Deltour, R., Jansen, A. G. M. & Wyder, P. Deviations from plastic barriers in $Bi_2Sr_2CaCu_2O_{8+\delta}$ thin films. *Phys. Rev. B* **71**, 052502 (2005).

[6] Wang, Y., Li, L. & Ong, N. P. Nernst effect in high-T_c superconductors. *Phys. Rev. B* **73**, 024510 (2006).

[7] Obertelli, S. D., Cooper, J. R. & Tallon, J. L. Systematics in the thermoelectric power of high-T_c oxides. *Phys. Rev. B* **46**, 14928(R) (1992).

[8] Munakata, F., Matsuura, K., Kubo, K., Kawano, T. & Yamauchi, H. Thermoelectric power of $Bi_2Sr_2Ca_{1-x}Y_xCu_2O_{8+y}$. *Phys. Rev. B* **45**, 10604 (1992).

[9] Cyr-Choinière, O., Daou, R., Laliberté, F., Collignon, C., Badoux, S., LeBoeuf, D., Chang, J., Ramshaw, B. J., Bonn, D. A., Hardy, W. N., Liang, R., Yan, J.-Q., Cheng, J.-G., Zhou, J.-S., Goodenough, J. B., Pyon, S., Takayama, T., Takagi, H., Doiron-Leyraud, N. & Taillefer, L. Pseudogap temperature T^* of cuprate superconductors from the Nernst effect. *Phys. Rev. B* **97**, 064502 (2018).

[10] Ussishkin, I., Sondhi, S. L., & Huse, D. A. Gaussian superconducting fluctuations, thermal transport, and the Nernst effect. *Phys. Rev. Lett.* **89**, 287001 (2002).

[11] Glatz, A., Pourret, A., & Varlamov, A. A. Analysis of the ghost and mirror fields in the Nernst signal induced by superconducting fluctuations. *Physical Review B*, **102**,

174507 (2020).

[12] Pourret, A., Aubin, H., Lesueur, J., Marrache-Kikuchi, C. A., Berge, L., Dumoulin, L. & Behnia, K. Observation of the Nernst signal generated by fluctuating Cooper pairs. *Nat. Phys.* **2**, 683-686 (2006).

[13] Chang, J., Doiron-Leyraud, N., Cyr-Choinière, O., Grissonnanche, G., Laliberté, F., Hassinger, E., Reid, J-Ph., Daou, R., Pyon, S., Takayama, T., Takagi, H. & Taillefer, L. Decrease of upper critical field with underdoping in cuprate superconductors. *Nat. Phys.* **8**, 751-756 (2012).

[14] Tafti, F. F., Laliberté, F., Dion, M., Gaudet, J., Fournier, P. & Taillefer, L. Nernst effect in the electron-doped cuprate superconductor $Pr_{2-x}Ce_xCuO_4$: Superconducting fluctuations, upper critical field H_{c2}, and the origin of the T_c dome. *Phys. Rev. B* **90**, 024519 (2014).

[15] Behnia, K. & Aubin. H. Nernst effect in metals and superconductors: a review of concepts and experiments. Rep. Prog. Phys. 79, 046502 (2016).

[16] Behnia, K. Fundamentals of Thermoelectricity (Oxford Univ. Press, 2015).

[17] Falson, J., Xu, Y., Liao, M., Zang, Y., Zhu, K., Wang, C., Zhang, Z., Liu, H., Duan, W., He, K., Liu, H., Smet, J. H., Zhang, D., & Xue, Q-. K. Type-II Ising pairing in few-layer stanene. *Science* **367**, 1454 (2020).

[18] Jacobs, T., Sridhar, S., Li, Q., Gu, G. D. & Koshizuka, N. In-plane and *c*-axis microwave penetration depth in $Bi_2Sr_2Ca_1Cu_2O_{8+\delta}$ crystals. *Phys. Rev. Lett.* **75**, 4516 (1995).

[19] Niderost, M., Frassanito, R., Saalfrank, M., Mota, A. C., Blatter, G., Zavaritsky, V. N., Li, T. W. & Kes, P. H. Lower critical field H_{c1} and barriers for vortex entry in $Bi_2Sr_2CaCu_2O_{8+\delta}$ crystals. *Phys. Rev. Lett.* **81**, 3231 (1998).

[20] Kogan, V. G., Ledvij, M., Simonov, A. Yu., Cho, J. H. & Johnston, D. C. Role of vortex fluctuations in determining superconducting parameters from magnetization data for layered superconductors. *Phys. Rev. Lett.* **70**, 1870 (1993).

[21] Lee, S. L., Zimmermann, P., Keller, H., Warden, M., Savic, I. M., Schauwecker, R., Zech, D., Cubitt, R., Forgan, E. M., Kes, P. H., Li, T. W., Menovsky, A. A. & Tarnawski,

Z. Evidence for flux-lattice melting and a dimensional crossover in single-crystal $Bi_{2.15}Sr_{1.85}CaCu_2O_{8+\delta}$ from muon spin rotation studies. *Phys. Rev. Lett.* **71**, 3862 (1993).

[22] Rischau, C. W., Li, Y., Fauqué, B., Inoue, H., Kim, M., Bell, C., Hwang, H. Y., Kapitulnik, A. & Behnia, K. Universal bound to the amplitude of the vortex Nernst signal in superconductors. *Phys. Rev. Lett.* 126, 077001 (2021).

[23] de Lange, O. L. & Otter, F. A. Flux flow effects in a nearly reversible type II superconductor. *J. Low Temp. Phys.* **18**, 31-42 (1975).

[24] Li, X. -Q., Li, Z. -L., Zhao, J. -J. & Wu, X. -S. Electrical and thermoelectric study of two-dimensional crystal of $NbSe_2$. *Chinese Phys. B* **29**, 087402 (2020).

[25] Logvenov, G. Y., Kartsovnik, M. V., Ito, H., & Ishiguro, T. Seebeck and Nernst effects in the mixed state of the two-band organic superconductors κ-(BEDT-TTF)$_2$Cu(NCS)$_2$ and κ-(BEDT-TTF)$_2$Cu[N(CN)$_2$]Br. *Met. Synth.* **86,** 2023 (1997).

[26] Pourret, A., Malone, L., Antunes, A. B., Yadav, C. S., Paulose, P. L., Fauqu´e, B. & Behnia, K. Strong correlation and low carrier density in $Fe_{1+y}Te_{0.6}Se_{0.4}$ as seen from its thermoelectric response. *Phys. Rev. B* **83**, 020504(R) (2011).

[27] Gollnik, F., & Naito, M. Doping dependence of normal-and superconducting-state transport properties of $Nd_{2-x}Ce_xCuO_{4\pm y}$ thin films. *Phys. Rev. B* **58**, 11734 (1998).

[28] Capan, C., Behnia, K., Hinderer, J., Jansen, A. G. M., Lang, W., Marcenat, C., Marin, C. & Flouquet, J. Entropy of vortex cores near the superconductor-insulator transition in an underdoped cuprate. *Phys. Rev. Lett.* **88**, 056601 (2002).

[29] Huebener, R. P. & Ri, H.-C. Vortex transport entropy in cuprate superconductors and Boltzmann constant. *Physica C* **591**, 1353975 (2021).

[30] Palstra, T. T. M., Batlogg, B., Schneemeyer, L. F. & Waszczak, J. V. Transport entropy of vortex motion in $YBa_2Cu_3O_7$. *Phys. Rev. Lett.* **64**, 3090 (1990).
